# Serum vascular endothelial growth factor is a potential biomarker for acute mountain sickness

**DOI:** 10.3389/fphys.2023.1083808

**Published:** 2023-03-30

**Authors:** Nasenien Nourkami-Tutdibi, Jennifer Küllmer, Sven Dietrich, Dominik Monz, Michael Zemlin, Erol Tutdibi

**Affiliations:** Saarland University Medical Center, Hospital for General Pediatrics and Neonatology, Homburg, Germany

**Keywords:** cytokines, biomarker, high altitude, hypobaric environment, hypobaric hypoxia, inflammation, acute mountain sickness

## Abstract

**Background:** Acute mountain sickness (AMS) is the most common disease caused by hypobaric hypoxia (HH) in high-altitude (HA) associated with high mortality when progressing to high-altitude pulmonary edema (HAPE) and/or high-altitude cerebral edema (HACE). There is evidence for a role of pro- and anti-inflammatory cytokines in development of AMS, but biological pathways and molecular mechanisms underlying AMS remain elusive. We aimed to measure changes in blood cytokine levels and their possible association with the development of AMS.

**Method:** 15 healthy mountaineers were included into this prospective clinical trial. All participants underwent baseline normoxic testing with venous EDTA blood sampling at the Bangor University in United Kingdom (69 m). The participants started from Beni at an altitude of 869 m and trekked same routes in four groups the Dhaulagiri circuit in the Nepali Himalaya. Trekking a 14-day route, the mountaineers reached the final HA of 5,050 m at the Hidden Valley Base Camp (HVBC). Venous EDTA blood sampling was performed after active ascent to HA the following morning after arrival at 5,050 m (HVBC). A panel of 21 cytokines, chemokines and growth factors were assessed using Luminex system (IL-1β, IL-2, IL-4, IL-6, IL-8, IL-10, IL-12p40, IL-1ra, sIL-2Rα, IFN-γ, TNF-α, MCP-1, MIP-1α, MIP-1β, IP-10, G-CSF, GM-CSF, EGF, FGF-2, VEGF, and TGF-β1).

**Results:** There was a significant main effect for the gradual ascent from sea-level (SL) to HA on nearly all cytokines. Serum levels for TNF-α, sIL-2Rα, G-CSF, VEGF, EGF, TGF-β1, IL-8, MCP-1, MIP-1β, and IP-10 were significantly increased at HA compared to SL, whereas levels for IFN-γ and MIP-1α were significantly decreased. Serum VEGF was higher in AMS susceptible *versus* AMS resistant subjects (*p* < 0.027, main effect of AMS) and increased after ascent to HA in both AMS groups (*p* < 0.011, main effect of HA). Serum VEGF increased more from SL values in the AMS susceptible group than in the AMS resistant group (*p* < 0.049, interaction effect).

**Conclusion:** Cytokine concentrations are significantly altered in HA. Within short interval after ascent, cytokine concentrations in HH normalize to values at SL. VEGF is significantly increased in mountaineers suffering from AMS, indicating its potential role as a biomarker for AMS.

## Introduction

Decades of research have been spent to investigate functions and limits of the human body at high-altitude (HA) and hypobaric hypoxia (HH) (Hackett and Roach, 2001; Wilson et al., 2009; West, 2015; Garrido et al., 2019). Research aimed for a better understanding of immunologic and metabolic response during HH and HA (Beidleman et al., 2018; Burtscher et al., 2021). Mountains like the Everest are very popular, touristic destinations with all its complications leading to pollution and a notable number of deaths every year (Firth et al., 2008; Pun, 2009). To prevent severe illness, mountaineers are advised to follow certain preparations and recommendations upfront travelling (Flaherty and Kennedy, 2016; Luks and Hackett, 2022). HA is characterized by a hypoxic and hypobaric environment, which can lead to cellular hypoxia and systemic inflammation through a modification of immune response (Pham et al., 2021). Acute mountain sickness (AMS) is a major threat to millions of people who live in or travel to HA. AMS is the most common disease caused by lower pressure and reduced oxygen amounts at HA (above 2,500 m) and can progress to high-altitude pulmonary edema (HAPE) and/or high-altitude cerebral edema (HACE) in severe cases associated with high mortality (Hackett and Roach, 2004; Luks et al., 2017). AMS development is the result of an interaction of several physiological responses to hypoxia while initiating several pathological processes (Lafuente J Bermudez et al., 2016; Lundeberg et al., 2018; Burtscher et al., 2022). Insufficient cerebrospinal compliance, alterations in fluid balance, activation of nociceptors induced by free radicals, and vasogenic edema induced by increased capillary permeability, have all been affiliated with AMS development. The biological pathways and exact molecular mechanisms underlying AMS remain elusive (Davis and Hackett, 2017; Li et al., 2018; Cobb et al., 2021). Cellular hypoxia, as well as cardiovascular and cerebrovascular responses all together influence the course of a variety of pulmonary and metabolic diseases and research within this field gives insights about mechanisms in the critically ill (Grocott et al., 2007; Heinonen et al., 2016). Understanding immunologic and metabolic responses during HA and HH may help to treat and/or prevent organic sequalae caused by HA and HH.

The influence of HH and HA on pro- and anti-inflammatory cytokines has been investigated thoroughly. Recent research supports the role of inflammatory processes in the development of AMS. Pro-inflammatory cytokines and other inflammatory markers like C-reactive protein (CRP) have been observed during short-term HA exposure (Hartmann et al., 2000; Lundeberg et al., 2018; Wang et al., 2018; Malacrida et al., 2019; Kammerer et al., 2020). Cytokines like IL-1ra, IL-1β, IL-6, IP-10, TNF-α, and transcription factors like nuclear factor kappa B (NF-κB) have been demonstrated to be part of physiological adjustments with changes in blood level during exposure to HA (Pedersen and Steensberg, 2002; Lundby and Steensberg, 2004; Liu et al., 2017a; Lundeberg et al., 2018). Inflammatory mediator expression appears to differ though, in individuals susceptible for AMS and those not susceptible for AMS. Those, developing AMS have been reported to show increased levels of acute phase proteins and inflammatory cytokines compared to non-AMS controls (Boos et al., 2016; Liu et al., 2017a; Wang et al., 2018). As a response to decreased partial oxygen pressures, hypoxia-inducible factor (HIF)-1α and HIF-2α are stabilized and dimerize with the nuclear HIF-1β subunit. This dimer interacts with hypoxia-response elements in promoter regions to increase expression of specific genes, for example, encoding erythropoietin and vascular endothelial growth factor (VEGF) (Semenza, 2012). Recent data indicated a strong association between VEGF and AMS susceptibility (Winter et al., 2021).

We hypothesize, that there is a significant influence of certain cytokines as VEGF in the development of AMS. The aim of the present study is to investigate changes of cytokines, chemokines, and growth factors in blood of healthy native lowlanders during a period of 2 weeks while trekking from sea-levels (SL) up to an altitude of 5050 m. We measure changes in blood to better understand cytokine regulation and their influence on the adaptation to a HA and HH and the development of AMS.

## Methods

### Participants and ascent profile

**FIGURE 1:**
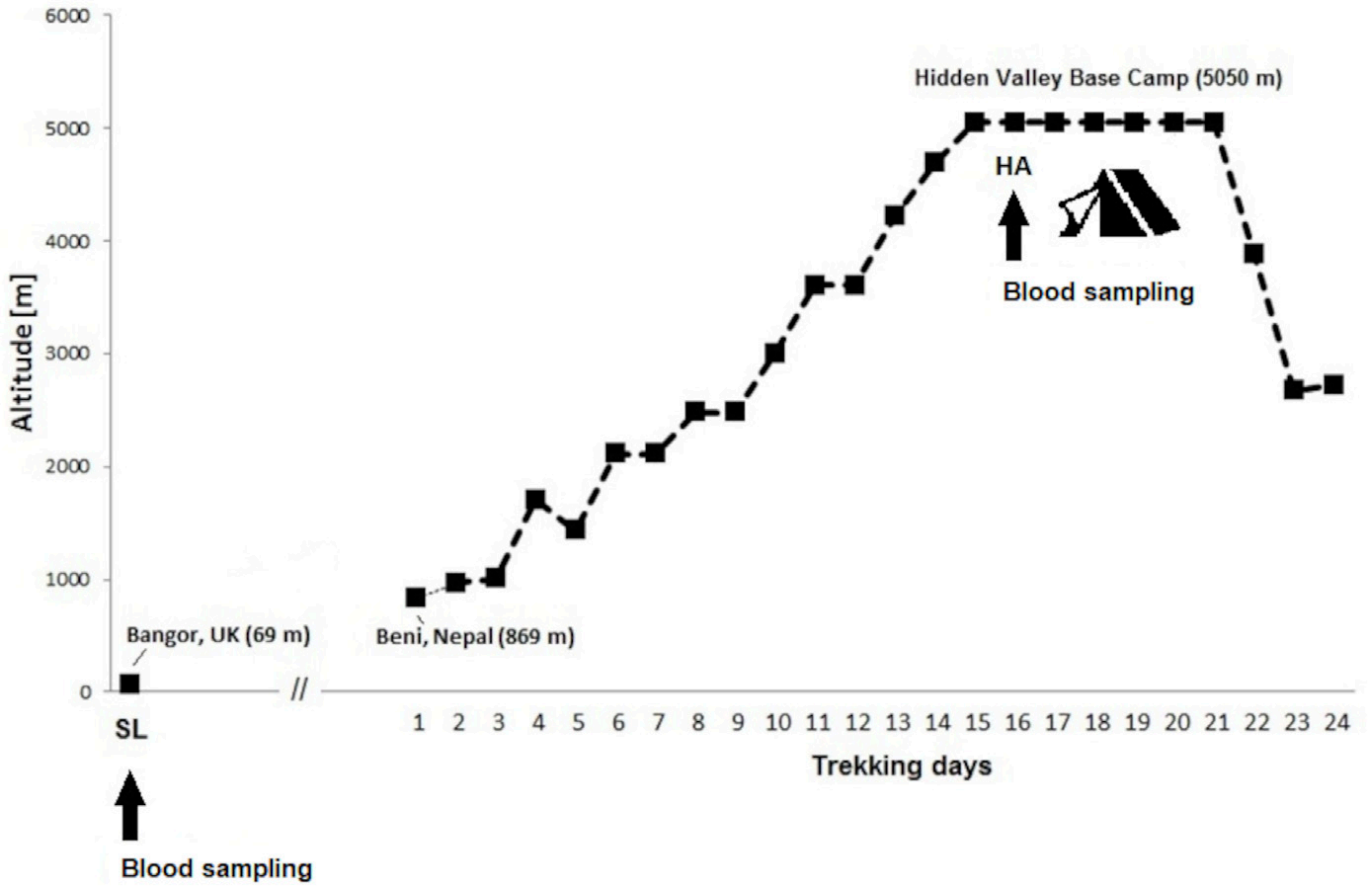
Ascent profile for Hidden Valley Base Camp. Data are sleeping altitudes for the expedition. Time points of blood sampling at sea-level (SL 69 m) in Bangor and at high-altitude (HA 5050 m) in the Hidden Valley Base Camp.

This observational prospective cohort study was conducted as part of the Hidden Valley Medical Expedition 2008. Healthy adults free from immunosuppression, cardiovascular, respiratory, renal, hepatic, neuromuscular or metabolic diseases were recruited for the current study. None of participants were under any regular medication. All participants were free to take any medication desired during expedition in Nepal. In total 15 healthy non-smokers and moderately active climbers were included for this study (6 female, nine male, age 34.5 ± 14.2 years, range 20–61). All study participants underwent baseline normoxic testing with blood sampling at the Bangor University in United Kingdom (69 m), from July to August 2008. To avoid hypoxic exposure, the participants remained below 2000 m altitude until the departure to Nepal in October 2008. The participants started the active ascent from Beni at an altitude of 869 m and trekked in four groups the same route from the Dhaulagiri circuit in the Nepali Himalaya. After a 14-day route, the mountaineers reached the final high-altitude of 5,050 m at the Hidden Valley Base Camp (HVBC). They remained there the following 6 days. Figure 1 illustrates timetable and expedition route.

### Blood sampling and processing

To assess plasma cytokines after active ascent to high-altitude (HA) blood samples were taken the following morning after arrival at 5,050 m (HVBC). Venous blood sampling was carried out according to the same standardized protocol at sea-level as well as at high-altitude. All blood samples were withdrawn from the study participants after overnight fasting and resting for 15 min in a sitting position. Samples were placed in EDTA tubes and centrifuged at 12,000 g for 10 min (MiniSpin, Eppendorf AG, Hamburg, Germany). Aliquots of obtained plasma were immediately frozen and kept continuously at −10°C or below in a portable freezer during the expedition and transport. On return to the United Kingdom, all samples obtained from the trials at SL and HA were shipped together to the research laboratory in Germany, where they were stored at −80°C and analysed within 3 months after expedition (January 2009).

### Ethical approval

Both the Coventry Ethics Committee and the Nepal Health Research Council approved the study. All participants provided written informed consent.

### Acute mountain sickness

Acute mountain sickness (AMS) was determined by the self-assessment questionnaires of the Lake Louise scoring system (LLS) during rest at SL and each morning throughout the expedition (Roach et al., 1993). AMS was diagnosed, when participants were above 2,500 m altitude and had LLS total score ≥3 with headache score ≥1. Those individuals without AMS over the expedition were classified as AMS resistant (AMS-) otherwise as AMS susceptible (AMS+), retrospectively. In addition, the percentage of trekking days with AMS, mean and total cumulative LLS scores were also calculated. LLS scores were used for correlations with serum cytokine concentrations.

### Blood count and peripheral oxygen saturation

Leucocyte counting was performed using the microscopic visualisation method in a Neubauer chamber. The concentration of haemoglobin was determined using the HemoCue Plasma Low Hb Analyser (Angelholm, Sweden). Haematocrit values were measured with a graphic reading device (Hawksley Microhematocrit Reader) after centrifugation (HaematoSpin 1,400, Hawksley, United Kingdom) of whole-blood samples in heparinized capillary tubes. Resting peripheral oxygen saturation (SpO2) was recorded in the mornings while seated for 10 min by means of a pulse oximeter (Onyx II 9550; Nonin, Minnesota).

### Cytokine analysis

The simultaneous quantification of cytokine levels in plasma was performed as described before on a Luminex 100 (Luminex Corporation, Austin, Texas, United States) with commercially available multiplex bead-based sandwich immunoassay kits. The Milliplex MPXHCYTO-60K assay (Millipore, Billerica, MA, United States) was used to quantify interleukin (IL)-1β, IL-1 receptor antagonist (IL-1ra), IL-2, soluble IL-2 receptor *α* (sIL-2Rα), IL-4, IL-6, IL-8, IL-10, IL-12p40, interferon *γ* (IFN-γ), interferon gamma inducible protein-10 (IP-10), monocyte chemoattractant protein-1 (MCP-1), macrophage inflammatory protein (MIP)-1α, MIP-1β, tumor necrosis factor *α* (TNF-α), granulocyte-macrophage colony-stimulating factor (GM-CSF), granulocyte colony-stimulating factor (G-CSF), epidermal growth factor (EGF), fibroblast growth factor (FGF-2), vascular endothelial growth factor (VEGF) and the TGFB-64K-01 to measure the transforming growth factor β1 (TGF-β1) according to the instructions provided by the manufacturer. A minimum of 50 beads per analyte was acquired. The readout of cytokine fluorescence intensities was analyzed with the standard version of the Milliplex Analyst software (Merck Millipore). A five-parameter logistic regression model was used to create standard curves and to calculate the marker concentration of each sample, expressed as pg/mL. Values of cytokine fluorescence intensities below background were set to missing. The lower limit of quantification (LLOQ) was set at the lowest standard concentration of 9.8 pg/mL for TGF-β1 and 3.2 pg/mL for other cytokines. For those samples whose cytokine concentration was detected below the LLOQ, the value was set at LLOQ for the statistical analysis. All Luminex measurements were performed on the same day in the laboratory of the Children’s University Hospital in Homburg/Saar, Germany. To minimize analytical variability all samples were analyzed as single samples in one-plate-run modus. An external pool control from healthy donors ran in duplicate to calculate the coefficient of variance (CV). The median intra-assay CV for the pooled replicate sample was 6.2% across all analytes (TNF-α = 2.9%, sIL-2Rα = 6.0%, IFN-γ = 12.6%, IL-1ra = 6.3%, G-CSF = 0.1%, VEGF = 9.7%, EGF = 10.3%, FGF-2 = 11.8%, TGF-β1 = 6.1%, IL-8 = 8.7%, MCP-1 = 10.0%, MIP-1α = 0%, MIP-1β = 2.9%, IP-10 = 3.2%).

### Statistical analysis

Statistical analysis was performed using IBM SPSS Statistics Version 19 (SPSS Inc, Chicago, IL, United States). All results are presented in numbers, rates and as mean ± standard deviations. Normal distribution of all continuous data was assessed by the Shapiro-Wilks test and inspection of the data by QQ-plots. Comparisons between AMS groups were assessed using the Fisher’s Exact test for categorical data and the unpaired *t*-test for continuous data. Paired *t*-test was used within the subjects to test for an effect of exposure to high-altitude. Comparisons of cytokine concentrations that were not normally distributed were followed by Box–Cox transformations to make the data distribution more normal. Differences between groups were assessed by a 2 × 2 mixed-model analysis of variance (ANOVA) using one dependent variable, one between-subject factor (AMS susceptibility) and one within-subject factor (ascent from sea-level to high-altitude). Correlation analyses were performed by calculating the Pearson’s coefficient of correlation. A value of *p* < 0.05 was considered statistically significant and no correction was performed for multiple testing. G*Power Version 3.1.3 was used to assess the effect size detectable given the sample size of 15 participants. Our study had a 80% power to detect effect sizes of Cohen’s f = 0.39 in the two-way repeated measures ANOVA using an F-Test with alpha of 0.05.

## Results

### Characteristics of participants

All subject’s baseline resting SpO2, and haematological parameters evaluated at SL were within normal limits. In total, 8 (53.3%) of 15 participants suffered at least once from clinically defined AMS during the entire expedition above 2,500 m altitude. None of mountaineers consumed drugs known to affect acclimatization such as acetazolamide or dexamethasone until arrival to the base camp at 5,050 m altitude. Neither participant’s age, gender nor body mass index (BMI), SpO2 and hematological values at sea-level were related to AMS susceptibility, days with AMS, mean and total cumulative LLS scores. The main characteristics and haematological markers of the mountaineers according AMS groups at SL and HA are presented in Table 1. Gender distribution and mean age were comparable between AMS resistant and AMS susceptible groups. Similarly, there was no difference in physiological and haematological parameters between AMS groups at SL as well as at HA. Active gradual ascent to the base camp at 5,050 m resulted in significant changes in BMI, SpO2 and haemoglobin values (all *p* < 0.001). As expected, we found a main effect for altitude, as SpO2 and BMI were lowered at high-altitude compared to sea-level (both *p* < 0.001), whereas there was no main effect for AMS susceptibility both on SpO2 (*p* < 0.27) and on BMI (*p* < 0.41). Interestingly, there was a statistically significant interaction between altitude and AMS susceptibility, as mean SpO2 was lowered more in AMS susceptible subjects by −5.1% ± 2.8% compared to AMS resistant subjects at HA (*p* < 0.031).

**TABLE 1:** Clinical characteristics of study participants at sea-level (Bangor) and 1 day after arrival at high-altitude in 5,050 m (Hidden Valley Base Camp, HVBC) according to AMS groups. Data are given as number [%] or mean ± standard deviation. *p < 0.01 for intra-group comparisons (SL vs. HA) tested by paired *t*-test. #inter-group comparison (AMS- vs. AMS+) at sea-level (SL) and at high-altitude (HA) using unpaired *t*-test test for continuous variables and Fisher exact test for categorical variables.

** **	**Total**	**AMS-**	**AMS+**	**P #**
**Number**	15 (100%)	7 (46.7%)	8 (53.3%)	AMS- vs. AMS+
**Gender** [m:f]	9:6	4:3	5:3	n.s.
**Age** [years]	34.5 ± 14.2	33.6 ± 17.2	35.4 ± 12.1	n.s.
**BMI** [kg/m2]				
*SL*	23.1 ± 2.7	22.4 ± 1.8	23.6 ± 3.4	n.s.
*HA*	22.3 ± 2.4*	21.8 ± 1.8*	22.8 ± 2.9*	n.s.
*diff [%]*	-3.0 ± 2.2	-2.8 ± 2.1	-3.2 ± 2.3	n.s.
**SpO2** [%]				
*SL*	96.4 ± 1.6	95.6 ± 1.5	97.1 ± 1.5	n.s.
*HA*	79.2 ± 5.8*	81.8 ± 5.7*	76.8 ± 5.1*	n.s.
*diff [%]*	-17.9 ± 6.2	-14.4 ± 5.9	-20.9 ± 5.0	<0.035
**Hb** [g/l]				
*SL*	146 ± 13	151 ± 12	142 ± 13	n.s.
*HA*	159 ± 21*	167 ± 25	154 ± 17*	n.s.
*diff [%]*	+9.3 ± 10.8	+10.2 ± 13.5	+8.4 ± 9.2	n.s.
**Hkt** [%]				
*SL*	41.4 ± 3.3	43.4 ± 3.3	40.3 ± 3.1	n.s.
*HA*	43.3 ± 4.1	44.6 ± 3.3	42.9 ± 4.2	n.s.
*diff [%]*	+4.9 ± 11.2	+1.5 ± 5.7	+6.4 ± 13.1	n.s.
**Leucocytes** [1000/µl]				
*SL*	3,347 ± 1,964	2,728 ± 2,356	3,887 ± 1,496	n.s.
*HA*	4,532 ± 2,443	4,204 ± 2,016	4,818 ± 2,872	n.s.
*diff [%]*	+79.0 ± 9.5	+129.6 ± 203.3	+34.8 ± 75.4	n.s.
**LLS** mean score	1.3 ± 1.1	0.5 ± 0.3	1.9 ± 1.2	<0.01
**LLS** total cumulative score	20.4 ± 19.4	7.9 ± 4.8	31.4 ± 20.2	<0.01
**Days with AMS** [%]	10.4 ± 13.6	0	19.5 ± 13.0	<0.02

### Cytokine levels

Nearly all of 21 tested cytokines, chemokines and growth factors were consistently detectable in the obtained samples except for FGF-2, which was not measurable in 8% of samples. No further analysis was done on following seven markers with values below LLOQ in more than 50% of samples: IL-1β (53%), IL-2 (83%), IL-4 (90%), IL-6 (60%), IL-10 (57%), IL-12p40 (60%) and GM-CSF (60%). Cytokine levels according to altitude of sampling and AMS groups are given in Table 2. At baseline, there was no difference in resting cytokine concentrations between AMS resistant and AMS susceptible groups. Furthermore, the baseline (normoxic) values of cytokines were not related to gender, age and BMI of study participants. The two-way mixed ANOVA revealed a significant main effect for the gradual ascent from SL to HA on nearly all cytokines. So, serum levels for TNF-α, sIL-2Rα, G-CSF, VEGF, EGF, TGF-β1, IL-8, MCP-1, MIP-1β and IP-10 were significantly enhanced at HA compared to SL, whereas levels for IFN-γ and MIP-1α were significantly reduced. In contrast, we found no significant main effect for AMS susceptibility or an interaction between altitude and AMS susceptibility on tested cytokines, except for VEGF. VEGF was higher in AMS susceptible *versus* AMS resistant subjects (*p* < 0.027, main effect of AMS) and increased after ascent to HA in both AMS groups (*p* < 0.011, main effect of high-altitude). VEGF increased more from SL values in AMS susceptible group than in AMS resistant group (*p* < 0.049, interaction effect). Finally, we found neither main effects of, nor interaction between high-altitude and AMS susceptibility for IL-1ra and FGF-2. Correlation analysis between and LLS scores, SpO2 and cytokine concentrations revealed a significant inverse association of SpO2 in the subgroup of individuals with AMS susceptibility (Table 3).

**TABLE 2:** Serum levels of cytokines (pg/ml) at sea-level (SL) and one day after arrival at high-altitude in 5,050 m (HA) according to AMS groups. Cytokine levels are given as mean ± standard deviation (with minimum and maximum values); *proportion of samples with values detected below the lower limit of quantification (LLOQ), biomarkers with values below LLOQ in >50% of samples were excluded from statistical analysis; **normally distributed (tested by QQ-plots and Shapiro-Wilk); P a) AMS- vs. AMS+ compared by unpaired t-test; #based on Box-Cox transformation; P altitude, significance level for main effect of altitude; P interaction, significance level for interaction between altitude and AMS susceptibility; P AMS, significance level for main effect of AMS susceptibility; n.s. not significant; SL sea-level, HA high-altitude.

			SL			HA				2x2 mixed-model ANOVA			
		<LLOQ*	AMS-	AMS+	P a)		AMS-	AMS+	P a)	F-value	P altitude	P AMS	P interaction
Pro-inflammatory	TNF-α	0%	8.6 ± 2.4 (6.6-13.8)	8.9 ± 4.8 (5.3-20.1)	n.s.		9.8 ± 3.3 (4.9-14.6)	17.1 ± 7.7 (7.5-28.4)	n.s.#	238.3#	<0.001	n.s.	n.s.
	sIL-2Rα	33%	59.9 ± 90.7 (3.2-253.2)	58.1 ± 144.9 (3.2-415.9)	n.s.		99.8 ± 85.8 (3.2-227.9)	222.0 ± 308.2 (3.2-931.4)	n.s.#	4.82#	<0.047	n.s.	n.s.
Th1	IFN-γ	10%	23.4 ± 22.5 (3.2-59.4)	20.8 ± 14.2 (3.2-45.1)	n.s.		14.3 ± 13.9 (3.2-41.8)	10.5 ± 4.4 (3.2-17.3)	n.s.#	5.09#	<0.042	n.s.	n.s.
Anti-inflammatory	IL-1ra	27%	35.6 ± 80.6 (3.2-218.1)	7.4 ± 7.9 (3.2-25.2)	n.s.		58.9 ± 140.9 (3.2-378.4)	37.2 ± 58.3 (5.9-176.9)	n.s.#	1.92#	n.s.	n.s.	n.s.
Growth factors	G-CSF	0%	40.5 ± 16.3 (25.4-64.7)	40.2 ± 17.2 (21.9-77.2)	n.s.		46.2 ± 13.7 (28.8-64.7)	90.6 ± 42.4 (42.1-161.3)	n.s.#	6,720#	<0.001	n.s.	n.s.
	VEGF**	0%	159.9 ± 226.4 (11.4-616.1)	305.4 ± 193.7 (16.0-529.6)	n.s.		196.6 ± 229.7 (11.4-472.4)	549.7 ± 210.7 (160.5-880.5)	<0.009	8.7	<0.011	<0.027	<0.049
	EGF	13%	12.4 ± 8.8 (3.2-25.9)	30.6 ± 44.7 (3.2-138.5)	n.s.		222.6 ± 412.9 (3.2-1,101)	751.4 ± 290.1 (290.1-1,060)	<0.011#	5.1#	<0.043	n.s.	n.s.
	FGF-2**	10%	90.2 ± 44.5 (41.5-177.6)	247.8 ± 333.0 (3.2-1,057)	n.s.		63.1 ± 49.6 (3.2-125.9)	251.4 ± 396.5 (25.3-1222.2)	n.s.	0.26	n.s.	n.s.	n.s.
	TGF-β1**	0%	3,857 ± 3,030 (678-9,265)	2,817 ± 2,321 (311-5,989)	n.s.		25,846 ± 27,827 (5,695-75,043)	40,705 ± 19,647 (294-60,845)	n.s.	24.3	<0.001	n.s.	n.s.
Chemokines	IL-8**	3%	5.1 ± 2.5 (3.6-10.9)	5.8 ± 2.0 (3.2-9.1)	n.s.		11.1 ± 7.3 (5.4-26.9)	11.7 ± 2.4 (7.7-14.6)	n.s.	14.9	<0.002	n.s.	n.s.
	MCP-1	0%	253.7 ± 67.4 (180.6-362.4)	244.8 ± 66.7 (174.8-384.5)	n.s.		389.3 ± 176.1 (221.7-632.9)	271.1 ± 74.4 (166.6-373.9)	n.s.#	155.3#	<0.001	n.s.	n.s.
	MIP-1α**	40%	29.6 ± 37.5 (3.2-98.5)	21.9 ± 18.6 (3.2-43.3)	n.s.		17.5 ± 19.3 (3.2-47.1)	14.8 ± 12.3 (3.2-31.7)	n.s.	5.6	<0.035	n.s.	n.s.
	MIP-1β	0%	25.0 ± 10.7 (9.7-41-5)	42.2 ± 31.2 (20.5-117.3)	n.s.		41.7 ± 13.4 (15.5-54.5)	100.2 ± 67.7 (51.2-238.8)	<0.011#	13.4#	<0.003	n.s.	n.s.
	IP-10	0%	773.8 ± 376.1 (402.1-1,560)	506.3 ± 105.7 (367.7-674.3)	n.s.		665.8 ± 153.9 (436.6-868.4)	683.3 ± 310.6 (375.9-1,268)	n.s.#	814.8#	<0.001	n.s.	n.s.

**TABLE 3:** Pearson’s correlation coefficients between cytokines and peripheral oxygen saturation (SpO2) by AMS groups.

	**Variables**	**SpO2 [%]**	
		**AMS-**	**AMS+**
**Pro-inflammatory**	TNF-α	−0.245	−0.575*
	sIL-2Rα	−0.346	−0.444
**Th1**	IFN-γ	0.138	0.463
**Anti-inflammatory**	IL-1ra	0.030	−0.478
**Growth factors**	G-CSF	−0.051	−0.519*
	VEGF	0.074	−0.605*
	EGF	−0.295	−0.841**
	FGF-2	0.182	−0.083
	TGF-β1	−0.212	−0.792**
**Chemokines**	IL-8	−0.171	−0.696**
	MCP-1	−0.164	−0.227
	MIP-1α	0.068	0.229
	MIP-1β	−0.391	−0.579*
	IP-10	0.233	−0.430

*p < 0.05 and **p < 0.01.

## Discussion

The development of AMS is a multifactorial incidence characterized by elevated cerebral blood flow, an imbalance in cerebral autoregulation and an increase in hypoxic mediators like nitrite oxide, prostaglandins, and VEGF (Hackett and Roach, 2001; Wilson et al., 2009). Some controversy exists about the underlying, mostly inflammatory, mechanisms of HH and its impact on a variety of immunologic processes (Halligan et al., 2016; Taylor and Colgan, 2017). Molecular mechanisms of HH like hypoxia-inducible factor signal cascade are well investigated (Semenza, 2006; Semenza, 2009). Hypoxia-induced inflammation can induce immune response resulting in a variety of pathologies in the context of chronic hypoxia (Walmsley et al., 2014; Krzywinska and Stockmann, 2018). The individual physiological response to HA hypoxia involves genetic and epigenetic factors besides condition and training (MacInnis et al., 2011; MacInnis et al., 2015; Childebayeva et al., 2021). AMS and its possible deterioration including HAPE and/or HACE is mainly caused by increased capillary pressure, i.e., a non-inflammatory phenomenon (Bärtsch et al., 2003; Bärtsch et al., 2005; Bärtsch and Saltin, 2008). VEGF is a hypoxia-induced protein inducing vascular permeability (Carmeliet et al., 1996; Ferrara et al., 1996; Ferrara et al., 2003; Weis and Cheresh, 2005). Evidence and research are still sparse about VEGF and its pathophysiologic role in the development of AMS.

### Changes in cytokines at hypobaric hypoxia

**FIGURE 2:**
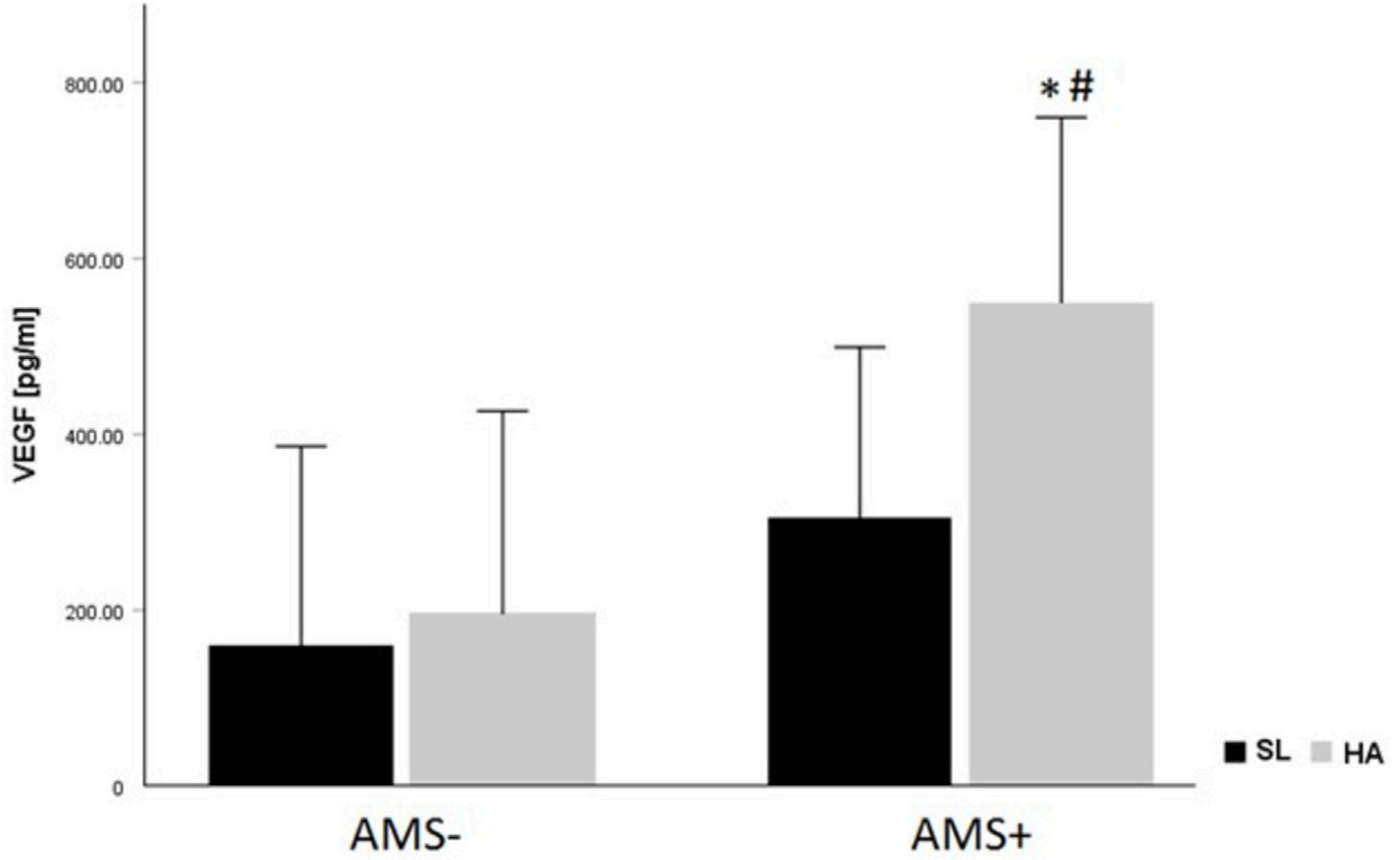
VEGF levels (pg/mL) according to altitude and AMS group. Data are given as mean ± standard deviation; *p < 0.05 for intra-group comparisons (SL vs. HA) tested by paired *t*-test. #*p* < 0.05 for inter-group comparison (AMS- vs. AMS+) at sea-level (SL) and at high-altitude (HA) using unpaired *t*-test.

In this study we compared and analyzed changes in cytokine levels of lowlanders at SL and HA with an emphasis on the development of AMS. In our study the cytokine levels at baseline showed no difference in resting cytokine concentrations between AMS resistant and AMS susceptible groups. Serum levels for TNF-α, sIL-2Rα, G-CSF, VEGF, EGF, TGF-β1, IL-8, MCP-1, MIP-1β, and IP-10 were significantly increased at HA compared to SL. In contrast to the latter, levels for IFN-γ and MIP-1α were significantly reduced. Baseline levels of cytokines were not related to gender, age, and BMI of the participating mountaineers. The two-way mixed ANOVA revealed a significant main effect for the gradual ascent from SL to HA on nearly all cytokines. The most significant finding of our study is, that VEGF was higher in AMS susceptible *versus* AMS resistant subjects and increased after ascent to HA in both AMS groups. VEGF increased more from SL values in AMS susceptible group than in AMS resistant group (Figure 2).

### Biomarkers for AMS

To investigate and study acclimatization in extreme environments, most studies are performed with native lowlanders ascending to HA under HH. AMS is experienced during ascent by many mountaineers and characterized by headache, nausea, fatigue, and gastrointestinal issues. The diagnosis depends on a self-questionnaire based on the LLS (Roach et al., 1993; Roach et al., 2018). Apart from a prior history of AMS, there are no routine clinical biomarkers to detect the susceptibility to AMS raising an urgent need for additional biomarkers for diagnosing and/or detection of patients susceptible to develop AMS. There is strong evidence for genetic factors and polymorphisms influencing HA adaptation (MacInnis et al., 2011; Buroker et al., 2012a; Yu et al., 2016; Zhang et al., 2020). There is also recent research focusing on transcriptome and miRNA analysis to identify potential biomarkers for AMS (Liu et al., 2017a; Liu et al., 2017b). Most research is performed on blood samples, but urine samples and urine metabolites as predictors for the development of AMS have been investigated recently as well (Richalet et al., 2012). Some studies aim to develop a risk prediction score to assess the likelihood for the development of AMS (Canouï-Poitrine et al., 2014; Lu et al., 2018). Longitudinal studies with multi-omics approach try to deal with the challenge to identify possible new biomarker in the diagnosis of AMS (Winter et al., 2021; Yang et al., 2022). One of the latter studies, performed by Lu et al., identified the VEGF signal pathway being suppressed in the AMS negative group after acute hypoxic exposure, but not in the AMS positive group. Copine-3, a crucial regulator of the VEGF signal pathway, is significantly decreased after exposure to hypoxia in individuals without symptoms of AMS in comparison to those suffering from AMS assuming that regulators of the VEGF signal pathway may contribute to the development of AMS.

### Data in scope of current research—VEGF and AMS

Our study is in good accordance with recent research (Walter et al., 2001). Some studies measured an increase of VEGF levels during ascent to HA without an association to AMS (Palma et al., 2006; Dorward et al., 2007), while other studies did not measure any changes in VEGF levels during ascent to HA (Maloney et al., 2000; Nilles et al., 2009; Schommer et al., 2011). Lower VEGF levels are known to be related with a lower susceptibility to symptoms related to AMS (Ding et al., 2011; Pham et al., 2021). The exact pathophysiological mechanisms, why some climbers are more susceptible to develop AMS while others remain unaffected throughout their ascent remains elusive. Genetic predisposition as polymorphisms in the VEGF-A gene may play an attributional role here (MacInnis et al., 2011; Rabie et al., 2011; Buroker et al., 2012b; Zhang et al., 2020). Possible VEGF upregulation through hypobaric, hypoxic pre-conditioning must be considered as well (Shi et al., 2001; Sen et al., 2011; Shao and Lu, 2012). Maintenance at SL without travel to HA environments for at least 1 month upfront to expedition was an essential inclusion criterion for all participants of our study. While interpreting higher VEGF levels it must be considered, that VEGF mRNA half-life (t1/2) extends under hypoxic circumstances up to 3.3 h affecting serum t1/2 VEGF serum levels (Shi et al., 2001; Liu et al., 2002). High-altitude VEGF levels measured in the non-AMS and AMS group are in very good accordance to recently published data from Winter et al. and early data from Tissot et al. (Tissot Van Patot et al., 2005; Winter et al., 2021). Both studies used enzyme-linked immunosorbent assay to analyze cytokine levels, whereas in our study we employed the Luminex method. Some studies analyzing cytokine levels in HA did not find any association between cytokine increase during ascent, nor did they find any association between cytokine levels and AMS (Nilles et al., 2009). In contrary to the latter, others did find an increase of VEGF levels at HA without an association to AMS though (Dorward et al., 2007; Patitucci et al., 2009; Schommer et al., 2011). Maloney et al. described a sustained VEGF plasma level at HA for those susceptible for AMS and a decrease of plasma VEGF in those mountaineers without AMS symptoms compared to plasma VEGF at SL (Maloney et al., 2000). Decreased plasma VEGF levels in healthy mountaineers without symptoms of AMS could be demonstrated by Oltmanns et al. as well (Oltmanns et al., 2006).

### Strengths and limitations of the study

One strength of our study is that none of our participants had been to HA within a month upfront to the expedition, as the rate of ascent and previous acclimatization may influence the development of AMS (Bärtsch and Saltin, 2008). Another strength of the study is that ascent profiles for all participant groups were the same and all participants climbed the same route. Blood was sampled at the same time after reaching a given altitude with control of variables such as caffeine, smoking, and eating before blood sampling. There are different types and rates of ascents in different published studies biasing data on VEGF level. As a strength of our study mountaineers climbed constantly above a certain threshold, allowing partial acclimatization. Despite low number of participants, gender distribution and mean age were comparable between AMS resistant and AMS susceptible groups and there was no difference in physiological and haematological parameters between AMS groups at SL as well as at HA. Main limitation of our study is the low number of participants due to the study design and location. Power calculation though reveals that the number of participants included in this study is sensitive to detect large effect sizes in cytokine profiles. An additional limitation of the current study is that serial blood sampling is missing for all participants to create a full cytokine profile from beginning of ascent until descent from HA and ideally 1–2 weeks after descent and acclimatization to SL.

## Conclusion

Hypoxia in HA is a potent trigger to influence serum cytokine concentrations. Studies demonstrating an association between cytokine levels and susceptibility to AMS are sparse. In this study serum VEGF was significantly increased in mountaineers suffering from AMS at 5,050 m, indicating its potential role as a biomarker for this condition. We were able to demonstrate a significant change and increase in VEGF serum levels in mountaineers suffering from AMS compared to the non-AMS group. As VEGF is the only cytokine being significantly increased, despite low sample size, demonstrates its potential role as a biomarker in the development of AMS.

## Data Availability

The raw data supporting the conclusion of this article will be made available by the authors, without undue reservation.

## Abbreviations

AMS, acute mountain sickness; BMI, body mass index; CRP C, reactive protein; CV, coefficient of variance; EDTA, ethylenediaminetetraacetic acid; EGF, epidermal growth factor; FGF-2, fibroblast growth factor-2; G-CSF, granulocyte colony-stimulating factor; GM-CSF, granulocyte-macrophage colony-stimulating factor; HA, high-altitude; HACE, high-altitude cerebral edema; HAPE, high-altitude pulmonary edema; HH, hypobaric hypoxia; HIF, hypoxia-inducible factor; HVBC, Hidden Valley Base Camp; IFN-γ,interferon γ; IL, interleukin; IL-1ra, interleukin-1 receptor antagonist; IP-10, interferon γ inducible protein-10; LLOQ, lower limit of quantification; LLS, Lake Louise score; MCP-1, monocyte chemoattractant protein-1; MIP-1, macrophage inflammatory protein-1; NF-κB, nuclear factor kappa B; sIL-2Rα, soluble IL-2 receptor α; SL, sea-level; SpO2, peripheral oxygen saturation; TGF-β1, transforming growth factor β1; TNF-α, tumor necrosis factor α; VEGF, vascular endothelial growth factor.

## References

[B1] Bärtsch P. Mairbäurl H. Maggiorini M. Swenson E. R. (2005). Physiological aspects of high-altitude pulmonary edema. J. Appl. Physiol. 98, 1101–1110. 10.1152/japplphysiol.01167.2004 15703168

[B2] Bärtsch P. Mairbäurl H. Swenson E. R. Maggiorini M. (2003). High altitude pulmonary oedema. Swiss Med. Wkly. 133, 377–384. 10.4414/smw.2003.09657 12947525

[B3] Bärtsch P. Saltin B. (2008). General introduction to altitude adaptation and mountain sickness. Scand. J. Med. Sci. Sports 18, 1–10. 10.1111/j.1600-0838.2008.00827.x 18665947

[B4] Beidleman B. A. Fulco C. S. Glickman E. L. Cymerman A. Kenefick R. W. Cadarette B. S. (2018). Acute mountain sickness is reduced following 2 days of staging during subsequent ascent to 4300m. High. Alt. Med. Biol. 19, 329–338. 10.1089/ham.2018.0048 30517038

[B5] Boos C. J. Woods D. R. Varias A. Biscocho S. Heseltine P. Mellor A. J. (2016). High altitude and acute mountain sickness and changes in circulating Endothelin-1, Interleukin-6, and Interleukin-17a. High. Alt. Med. Biol. 17, 25–31. 10.1089/ham.2015.0098 26680502

[B6] Buroker N. E. Ning X. H. Zhou Z. N. Li K. Cen W. J. Wu X. F. (2012). AKT3, ANGPTL4, eNOS3, and VEGFA associations with high altitude sickness in Han and Tibetan Chinese at the Qinghai-Tibetan plateau. Int. J. Hematol. 96, 200–213. 10.1007/s12185-012-1117-7 22729570

[B7] Buroker N. E. Ning X. H. Zhou Z. N. Li K. Cen W. J. Wu X. F. (2012). EPAS1 and EGLN1 associations with high altitude sickness in Han and Tibetan Chinese at the Qinghai-Tibetan Plateau. Blood Cells Mol. Dis. 49, 67–73. 10.1016/j.bcmd.2012.04.004 22595196

[B8] Burtscher J. Mallet R. T. Pialoux V. Millet G. P. Burtscher M. (2022). Adaptive responses to hypoxia and/or hyperoxia in humans. Antioxid. Redox Signal 37, 887–912. 10.1089/ars.2021.0280 35102747

[B9] Burtscher M. Hefti U. Hefti J. P. (2021). High-altitude illnesses: Old stories and new insights into the pathophysiology, treatment and prevention. Sports Med. Health Sci. 3, 59–69. 10.1016/j.smhs.2021.04.001 35782163 PMC9219347

[B10] Canouï-Poitrine F. Veerabudun K. Larmignat P. Letournel M. Bastuji-Garin S. Richalet J. P. (2014). Risk prediction score for severe high altitude illness: A cohort study. PLoS One 9, 100642. 10.1371/journal.pone.0100642 PMC4113313 25068815

[B11] Carmeliet P. Ferreira V. Breier G. Pollefeyt S. Kieckens L. Gertsenstein M. (1996). Abnormal blood vessel development and lethality in embryos lacking a single VEGF allele. Nature 380, 435–439. 10.1038/380435a0 8602241

[B12] Childebayeva A. Harman T. Weinstein J. Day T. Brutsaert T. D. Bigham A. W. (2021). Genome-wide DNA methylation changes associated with high-altitude acclimatization during an everest base camp trek. Front. Physiol. 12, 660906. 10.3389/fphys.2021.660906 34262470 PMC8273439

[B13] Cobb A. B. Levett D. Z. H. Mitchell K. Aveling W. Hurlbut D. Gilbert-Kawai E. (2021). Physiological responses during ascent to high altitude and the incidence of acute mountain sickness. Physiol. Rep. 9, 14809. 10.14814/phy2.14809 PMC8077104 33904650

[B14] Davis C. Hackett P. (2017). Advances in the prevention and treatment of high altitude illness. Emerg. Med. Clin. North Am. 35, 241–260. 10.1016/j.emc.2017.01.002 28411926

[B15] Ding H. Liu Q. Hua M. Ding M. Du H. Zhang W. (2011). Polymorphisms of hypoxia-related genes in subjects susceptible to acute mountain sickness. Respiration 81, 236–241. 10.1159/000322850 21242666

[B16] Dorward D. A. Roger Thompson A. A. Kenneth Baillie J. MacDougall M. Hirani N. (2007). Change in plasma vascular endothelial growth factor during onset and recovery from acute mountain sickness. Respir. Med. 101, 587–594. 10.1016/j.rmed.2006.06.014 16890420

[B17] Ferrara N. Carver-Moore K. Chen H. Dowd M. Lu L. O’Shea K. S. (1996). Heterozygous embryonic lethality induced by targeted inactivation of the VEGF gene. Nature 380, 439–442. 10.1038/380439a0 8602242

[B18] Ferrara N. Gerber H. P. LeCouter J. (2003). The biology of VEGF and its receptors. Nat. Med. 9, 669–676. 10.1038/nm0603-669 12778165

[B19] Firth P. G. Zheng H. Windsor J. S. Sutherland A. I. Imray C. H. Moore G. W. K. (2008). Mortality on mount everest, 1921-2006: Descriptive study. BMJ 337, 2654. 10.1136/bmj.a2654 PMC2602730 19074222

[B20] Flaherty G. T. Kennedy K. M. (2016). Preparing patients for travel to high altitude: Advice on travel health and chemoprophylaxis. Br. J. General Pract. 66, e62–e64. 10.3399/bjgp16X683377 PMC4684038 26719484

[B21] Garrido E. Soria J. M. Salisbury R. (2019). Breathless and dying on mount everest. Lancet Respir. Med. 7, 938–939. 10.1016/S2213-2600(19)30281-4 31645281

[B22] Grocott M. Montgomery H. Vercueil A. (2007). High-altitude physiology and pathophysiology: Implications and relevance for intensive care medicine. Crit. Care 11, 203. 10.1186/cc5142 17291330 PMC2151873

[B23] Hackett P. H. Roach R. C. (2004). High altitude cerebral edema. High. Alt. Med. Biol. 5, 136–146. 10.1089/1527029041352054 15265335

[B24] Hackett P. H. Roach R. C. (2001). High-altitude illness. N. Engl. J. Med. 345, 107–114. 10.1056/NEJM200107123450206 11450659

[B25] Halligan D. N. Murphy S. J. E. Taylor C. T. (2016). The hypoxia-inducible factor (HIF) couples immunity with metabolism. Semin. Immunol. 28, 469–477. 10.1016/j.smim.2016.09.004 27717536

[B26] Hartmann G. Tschöp M. Fischer R. Bidlingmaier C. Riepl R. Tschöp K. (2000). High altitude increases circulating interleukin-6, interleukin-1 receptor antagonist and C-reactive protein. Cytokine 12, 246–252. 10.1006/cyto.1999.0533 10704252

[B27] Heinonen I. H. A. Boushel R. Kalliokoski K. K. (2016). The circulatory and metabolic responses to hypoxia in humans - with special reference to adipose tissue physiology and obesity. Front. Endocrinol. (Lausanne) 7, 116. 10.3389/fendo.2016.00116 27621722 PMC5002918

[B28] Kammerer T. Faihs V. Hulde N. Stangl M. Brettner F. Rehm M. (2020). Hypoxic-inflammatory responses under acute hypoxia: *In vitro* experiments and prospective observational expedition trial. Int. J. Mol. Sci. 21, 1034. 10.3390/ijms21031034 32033172 PMC7037641

[B29] Krzywinska E. Stockmann C. (2018). Hypoxia, metabolism and immune cell function. Biomedicines 6, 56. 10.3390/biomedicines6020056 29762526 PMC6027519

[B30] Li Y. Zhang Y. Zhang Y. (2018). Research advances in pathogenesis and prophylactic measures of acute high altitude illness. Respir. Med. 145, 145–152. 10.1016/j.rmed.2018.11.004 30509704

[B31] Liu B. Chen J. Zhang L. Gao Y. Cui J. Zhang E. (2017). IL-10 dysregulation in Acute Mountain sickness revealed by transcriptome analysis. Front. Immunol. 8, 628. 10.3389/fimmu.2017.00628 28611780 PMC5447681

[B32] Liu B. Huang H. Wu G. Xu G. Sun B. D. Zhang E. L. (2017). A signature of circulating microRNAs predicts the susceptibility of acute mountain sickness. Front. Physiol. 8, 55. 10.3389/fphys.2017.00055 28228730 PMC5296306

[B33] Liu L. X. Lu H. Luo Y. Date T. Belanger A. J. Vincent K. A. (2002). Stabilization of vascular endothelial growth factor mRNA by hypoxia-inducible factor 1. Biochem. Biophys. Res. Commun. 291, 908–914. 10.1006/bbrc.2002.6551 11866451

[B34] Lu H. Wang R. Li W. Xie H. Wang C. Hao Y. (2018). Plasma proteomic study of acute mountain sickness susceptible and resistant individuals. Sci. Rep. 8, 1265–1269. 10.1038/s41598-018-19818-9 29352170 PMC5775437

[B35] Luks A. M. Hackett P. H. (2022). Medical conditions and high-altitude travel. N. Engl. J. Med. 386, 364–373. 10.1056/nejmra2104829 35081281

[B36] Luks A. M. Swenson E. R. Bärtsch P. (2017). Acute high-altitude sickness. Eur. Respir. Rev. 26, 160096. 10.1183/16000617.0096-2016 28143879 PMC9488514

[B37] Lundby C. Steensberg A. (2004). Interleukin-6 response to exercise during acute and chronic hypoxia. Eur. J. Appl. Physiol. 91, 88–93. 10.1007/s00421-003-0935-y 12955521

[B38] Lundeberg J. Feiner J. R. Schober A. Sall J. W. Eilers H. Bickler P. E. (2018). Increased cytokines at high altitude: Lack of effect of ibuprofen on Acute Mountain sickness, physiological variables, or cytokine levels. High. Alt. Med. Biol. 19, 249–258. 10.1089/ham.2017.0144 29924642

[B39] MacInnis M. J. Wang P. Koehle M. S. Rupert J. L. (2011). The genetics of altitude tolerance: The evidence for inherited susceptibility to acute mountain sickness. J. Occup. Environ. Med. 53, 159–168. 10.1097/JOM.0b013e318206b112 21270658

[B40] MacInnis M. J. Widmer N. Timulsina U. Subedi A. Siwakoti A. Pandit B. P. (2015). A preliminary genome-wide association study of Acute Mountain sickness susceptibility in a group of Nepalese pilgrims ascending to 4380 m. High. Alt. Med. Biol. 16, 290–297. 10.1089/ham.2015.0065 26600424

[B41] Malacrida S. Giannella A. Ceolotto G. Reggiani C. Vezzoli A. Mrakic-Sposta S. (2019). Transcription factors regulation in human peripheral white blood cells during hypobaric hypoxia exposure: An *in-vivo* experimental study. Sci. Rep. 9, 9901. 10.1038/s41598-019-46391-6 31289332 PMC6617471

[B42] Maloney J. Wang D. Duncan T. Voelkel N. Ruoss S. (2000). Plasma vascular endothelial growth factor in acute mountain sickness. Chest 118, 47–52. 10.1378/chest.118.1.47 10893358

[B43] Nilles E. Sayward H. D’Onofrio G. (2009). Vascular endothelial growth factor and acute mountain sickness. J. Emerg. Trauma Shock 2, 6–9. 10.4103/0974-2700.44675 19561948 PMC2700574

[B44] Oltmanns K. M. Gehring H. Rudolf S. Schultes B. Hackenberg C. Schweiger U. (2006). Acute hypoxia decreases plasma VEGF concentration in healthy humans. Am. J. Physiol. Endocrinol. Metab. 290, E434–E439. 10.1152/ajpendo.00508.2004 16219663

[B45] Palma J. Macedonia C. Deuster P. Olsen C. Mozayeni B. R. Crutchfield K. E. (2006). Cerebrovascular dynamics and vascular endothelial growth factor in acute mountain sickness. Wilderness Environ. Med. 17, 1–7. 10.1580/1080-6032(2006)17[1:CDAVEG]2.0.CO;2 16538938

[B46] Patitucci M. Lugrin D. Pagès G. (2009). Angiogenic/lymphangiogenic factors and adaptation to extreme altitudes during an expedition to Mount Everest. Acta Physiol. 196, 259–265. 10.1111/j.1748-1716.2008.01915.x 18983460

[B47] Pedersen B. K. Steensberg A. (2002). “Exercise and hypoxia: Effects on leukocytes and interleukin-6 - shared mechanisms?” in Medicine and Science in Sports and Exercise (Indianapolis, IN, USA: American College of Sports Medicine), 2004–2012. 10.1097/00005768-200212000-00022 12471309

[B48] Pham K. Parikh K. Heinrich E. C. (2021). Hypoxia and inflammation: Insights from high-altitude physiology. Front. Physiol. 12, 676782. 10.3389/fphys.2021.676782 34122145 PMC8188852

[B49] Pun M. (2009). Important points in analysing deaths on Mount Everest. BMJ (Online) 338, b41. 10.1136/bmj.b41 19136543

[B50] Rabie T. Kunze R. Marti H. H. (2011). Impaired hypoxic response in senescent mouse brain. Int. J. Dev. Neurosci. 29, 655–661. 10.1016/j.ijdevneu.2011.06.003 21704147

[B51] Richalet J-P. Larmignat P. Poitrine E. Letournel M. Canouï-Poitrine F. (2012). Physiological risk factors for severe high-altitude illness: A prospective cohort study. Am. J. Respir. Crit. Care Med. 185, 192–198. 10.1164/rccm.201108-1396OC 22071330

[B52] Roach R. C. B. P. H. P. and O. O. (1993). “The Lake louise Acute Mountain sickness scoring system,” in Hypoxia and molecular medicine sutton JR. Editors Houston C. S. Coates G. (Burlington, NJ, USA: Queen City Press), 272–274.

[B53] Roach R. C. Hackett P. H. Oelz O. Bärtsch P. Luks A. M. MacInnis M. J. (2018). The 2018 lake louise acute mountain sickness score. High. Alt. Med. Biol. 19, 4–6. 10.1089/ham.2017.0164 29583031 PMC6191821

[B54] Schommer K. Wiesegart N. Dehnert C. Mairbäurl H. Bärtsch P. (2011). No correlation between plasma levels of vascular endothelial growth factor or its soluble receptor and acute mountain sickness. High. Alt. Med. Biol. 12, 323–327. 10.1089/ham.2011.1020 22206557

[B55] Semenza G. L. (2012). Hypoxia-inducible factors in physiology and medicine. Cell 148, 399–408. 10.1016/j.cell.2012.01.021 22304911 PMC3437543

[B56] Semenza G. L. (2009). Regulation of oxygen homeostasis by hypoxia-Inducible factor 1. Physiology 24, 97–106. 10.1152/physiol.00045.2008 19364912

[B57] Semenza G. L. (2006). “Regulation of physiological responses to continuous and intermittent hypoxia by hypoxia-inducible factor 1,” in Experimental Physiology (Hoboken, NJ, USA: Blackwell Publishing Ltd), 803–806. 10.1113/expphysiol.2006.033498 16740642

[B58] Sen E. Basu A. Willing L. B. Uliasz T. F. Myrkalo J. L. Vannucci S. J. (2011). Pre-conditioning induces the precocious differentiation of neonatal astrocytes to enhance their neuroprotective properties. ASN Neuro 3, 62–170. 10.1042/AN20100029 PMC3153963 21722095

[B59] Shao G. Lu G. W. (2012). Hypoxic preconditioning in an autohypoxic animal model. Neurosci. Bull. 28, 316–320. 10.1007/s12264-012-1222-x 22622832 PMC5560319

[B60] Shi Q. Le X. Wang B. Abbruzzese J. L. Xiong Q. He Y. (2001). Regulation of vascular endothelial growth factor expression by acidosis in human cancer cells. Oncogene 20, 3751–3756. 10.1038/sj.onc.1204500 11439338

[B61] Taylor C. T. Colgan S. P. (2017). Regulation of immunity and inflammation by hypoxia in immunological niches. Nat. Rev. Immunol. 17, 774–785. 10.1038/nri.2017.103 28972206 PMC5799081

[B62] Tissot Van Patot M. C. Leadbetter G. Keyes L. E. Bendrick-Peart J. Beckey V. E. Christians U. (2005). Greater free plasma VEGF and lower soluble VEGF receptor-1 in acute mountain sickness. J. Appl. Physiol. 98, 1626–1629. 10.1152/japplphysiol.00589.2004 15649874

[B63] Lafuente J v. Bermudez G. Camargo-Arce L. Bulnes S. (2016). Blood-Brain barrier changes in high altitude. CNS Neurol. Disord. Drug Targets 15, 1188–1197. 10.2174/1871527315666160920123911 27667557

[B64] Walmsley S. Harris A. Thompson A. A. R. MoiraKB Whyte (2014). HIF-Mediated innate immune responses: Cell signaling and therapeutic implications. Hypoxia 2, 47–58. 10.2147/hp.s50269 27774466 PMC5045056

[B65] Walter R. Maggiorini M. Scherrer U. Contesse J. Reinhart W. H. (2001). Effects of high-altitude exposure on vascular endothelial growth factor levels in man. Eur. J. Appl. Physiol. 85, 113–117. 10.1007/s004210100419 11513303

[B66] Wang C. Jiang H. Duan J. Chen J. Wang Q. Liu X. (2018). Exploration of acute phase proteins and inflammatory cytokines in early stage diagnosis of Acute Mountain sickness. High. Alt. Med. Biol. 19, 170–177. 10.1089/ham.2017.0126 29608374

[B67] Weis S. M. Cheresh D. A. (2005). Pathophysiological consequences of VEGF-induced vascular permeability. Nature 437, 497–504. 10.1038/nature03987 16177780

[B68] West J. B. (2015). High-altitude medicine. Lancet Respir. Med. 3, 12–13. 10.1016/S2213-2600(14)70238-3 25466336

[B69] Wilson M. H. Newman S. Imray C. H. (2009). The cerebral effects of ascent to high altitudes. Lancet Neurol. 8, 175–191. 10.1016/S1474-4422(09)70014-6 19161909

[B70] Winter C. Bjorkman T. Miller S. Nichols P. Cardinal J. O’Rourke P. (2021). Acute Mountain sickness following incremental trekking to high altitude: Correlation with plasma vascular endothelial growth factor levels and the possible effects of dexamethasone and acclimatization following Re-exposure. Front. Physiol. 12, 746044. 10.3389/fphys.2021.746044 34744786 PMC8567072

[B71] Yang J. Jia Z. Song X. Shi J. Wang X. Zhao X. (2022). Proteomic and clinical biomarkers for acute mountain sickness in a longitudinal cohort. Commun. Biol. 5, 548–614. 10.1038/s42003-022-03514-6 35668171 PMC9170681

[B72] Yu J. Zeng Y. Chen G. Bian S. Qiu Y. Liu X. (2016). Analysis of high-altitude syndrome and the underlying gene polymorphisms associated with Acute Mountain sickness after a rapid ascent to high-altitude. Sci. Rep. 6, 38323. 10.1038/srep38323 27982053 PMC5159877

[B73] Zhang J. H. Shen Y. Liu C. Yang J. Yang Y. Q. Zhang C. (2020). EPAS1 and vegfa gene variants are related to the symptoms of acute mountain sickness in Chinese han population: A cross-sectional study. Mil. Med. Res. 7, 35. 10.1186/s40779-020-00264-6 32718338 PMC7385974

